# The impact of sirolimus therapy on lesion size, clinical symptoms, and quality of life of patients with lymphatic anomalies

**DOI:** 10.1186/s13023-019-1118-1

**Published:** 2019-06-13

**Authors:** Michio Ozeki, Akifumi Nozawa, Shiho Yasue, Saori Endo, Ryuta Asada, Hiroya Hashimoto, Toshiyuki Fukao

**Affiliations:** 1 Department of Pediatrics, Graduate School of Medicine, Gifu University, 1-1, Yanagido, Gifu, 501-1194 Japan; 2 Innovative and Clinical Research Promotion Center, Graduate School of Medicine, Gifu University, 1-1, Yanagido, Gifu, 501-1194 Japan; 3 Clinical Research Center, National Hospital Organization Nagoya Medical Center, 4-1-1, Sannomaru, Naka-ku, Nagoya, Aichi 460-0001 Japan

**Keywords:** Vascular malformations, Lymphatic malformation, Generalized lymphatic anomaly, Gorham-stout disease, Mammalian target of rapamycin

## Abstract

**Background:**

Lymphatic anomalies (LAs) include several disorders in which abnormal lymphatic tissue invades the neck, chest, and various organs. Progressive cases may result in lethal outcomes and have proven difficult to treat. Sirolimus is showing promising results in the management of vascular anomalies. We examined the efficacy and safety of sirolimus treatment in patients with progressive LAs.

**Methods:**

All patients with LAs treated with sirolimus from May 2015 to September 2018 were included. They received oral sirolimus once a day and the dose was adjusted so that the trough concentration remained within 5–15 ng/mL. We prospectively reviewed the response to drugs (the response rate of radiological volumetric change of the target lesion), severity scores, reported quality of life (QOL), and adverse effects at 6 months after administration.

**Results:**

Twenty patients (five with cystic lymphatic malformation (LM), three with kaposiform lymphangiomatosis, three with generalized lymphatic anomaly, six with Gorham-Stout disease, and three with central conducting lymphatic anomaly) were treated with sirolimus at our institution. Fifty percent of patients (10/20) demonstrated a partial response by a radiological examination and a significant improvement in disease severity and QOL scores (*P* = 0.0020 and *P* = 0.0117, respectively). Ten patients who had no reduction in lesion size (stable disease group) showed no significant improvement in disease severity and QOL scores. Eighty percent of patients (16/20) had side effects, such as stomatitis, infection, and hyperlipidemia.

**Conclusions:**

Sirolimus impacts the reduction of the lymphatic tissue volume of LMs and could lead to improvement in clinical symptoms and QOL.

**Trial registration:**

UMIN Clinical Trials Registry, UMIN000016580. Registered 19 February 2015,

**Electronic supplementary material:**

The online version of this article (10.1186/s13023-019-1118-1) contains supplementary material, which is available to authorized users.

## Background

Lymphatic anomalies (LAs) are rare diseases that are caused by abnormalities of the lymphatic system and include cystic lymphatic malformation (LM), generalized lymphatic anomaly (GLA), Gorham-Stout disease (GSD), and central conducting lymphatic anomaly (CCLA) [1]. These are classified as LMs, according to the International Society for the Study of Vascular Anomalies (ISSVA) classification [2]. Cystic LMs present at birth in up to 60% of LA cases and the pathology of cystic LMs is quite variable, ranging from a focal area with minimal swelling to large areas of diffusely infiltrating aberrant lymphatic channels [3]. Depending on their location and extent of the lesion, LMs can affect vital physiological functions. Surgical resection and sclerotherapy are usually effective in treating and resolving macrocystic LMs. However, microcystic LMs are more infiltrative and difficult to treat. Interferon, corticosteroids, and propranolol have been used in the treatment of inoperable LMs, although their effects are limited and they are not indicated for this disease [3]. GLA is a rare and often fatal congenital lymphatic disorder that also commonly affects bone; it occurs predominantly in childhood or early adulthood. Kaposiform lymphangiomatosis (KLA) is a novel subtype of GLA with foci of spindle endothelial cells amid a background of malformed lymphatic channels [1, 4]. GSD is also characterized by lymphatic malformation, affecting a single or multiple bones and adjacent soft tissues; the osteolysis is progressive and invades the bone cortex [4]. The progression of GSD often includes visceral progression with thoracic and abdominal involvement, leading to effusions and ascites [4]. CCLAs are channel-type lymphatic malformations of named trunks. Anatomic changes like stenosis or closure of the large draining lymphatics like the thoracic duct as well as dysfunction lead to a reflux into the conducting channels with leakage into organs and skin [1]. A survey of previously published studies [4] of these diseases in Japan showed that the mortality rate was 20% (17/85) and all of the deceased had thoracic lesions. Management of LAs is challenging, with frequent poor responses to medical therapy and a poor prognosis [1].

The mammalian target of rapamycin (mTOR) activates protein synthesis, resulting in numerous cellular processes including cell proliferation and increased angiogenesis, thus playing a key role in the pathogenesis of various vascular anomalies [5]. The mTOR inhibitor sirolimus has been identified recently as a promising treatment for LAs. Hammill et al. reported on four patients with diffuse microcystic LM who displayed good clinical response to sirolimus treatment with mild and reversible side effects [5]. Adams et al. showed a high response rate to sirolimus treatment in LA patients [6]. Some studies have investigated the mechanisms by which sirolimus acts on lymphatic endothelial cells and LM lesions in these patients [7]. Sirolimus has also been shown to inhibit lymphangiogenesis [7]. In preclinical models, rapamycin prevents or inhibits lymphangiogenesis in zebrafish [8] and in mouse skin flaps, kidney allograft injury, and tumor metastasis [9, 10]. Rapamycin also suppresses growth of lymphatic endothelial cells in vitro [11]. It is thought to act on lymphatic tissues within lesions to regulate the production and leakage of lymph by decreasing lymphatic endothelial cell activity. Thus, we conducted this study to verify whether treatment with sirolimus will reduce the volume of lesions in lymphatic tissues and improve clinical symptoms in patients with LAs.

Herein we present our experience with 20 patients treated with sirolimus and discuss medical actions taken in the treatment of lymphatic lesions and patient outcomes.

## Methods

### Study objectives

The primary objective of the study was to assess the radiographic response rate (response rate of radiological volumetric change of the target lesion) to sirolimus treatment at 6 months. Secondary study objectives were as follows:

- ► To assess the radiological response rate at 3 months
- ► To assess improvement in the clinical severity score and the quality of life (QOL) score at 6 months
- ► To assess the association of the radiological response with improvement in the clinical severity and QOL scores
- ► To assess the safety (adverse events and side effects) of sirolimus treatment in patients with LAs

### Study outline and enrollment

This was a prospective trial at Gifu University Hospital in Japan. Informed consent was obtained from patients, parents, or legal guardians (when the subject’s age was less than 20 years at consent). Inclusion criteria were as follows: definitively diagnosed with cystic LM (head, neck, thoracic, peritoneal cavity, or retroperitoneum), GLA, GSD, or CCLA, excluding other lymphatic diseases (primary lymphedema and others); having at least one target lesion (e.g., cystic LM or lymphedema) that was measurable using magnetic resonance imaging (MRI); and having severe disorders and symptoms that required systemic therapy because of the target disease (bleeding, chronic pain, recurrent cellulitis, ulceration, visceral and/or bone involvement, as well as potential effects on organ function, including the eye, airway, and ear). Criteria for patients with LAs were defined as follows: cystic LM involved single, or multiple cystic LM lesions; KLA also involved multiple lymphatic lesions with spindle cell foci confirmed by pathological examination; GSD involved cortical bone loss and/or progressive bone resorption; GLA involved diffuse multiple lymphatic lesions in which spindle cell foci were not confirmed by pathological examination and there was an absence of progressive osteolysis; and CCLA had evidence of central conducting lymphatic channel abnormalities by radiological examination. Exclusion criteria were as follows: an active infection that requires systemic treatment; uncontrolled diabetes, hypertension, hyperlipidemia, or a chronic liver or kidney disease; a history of an allergic reaction to sirolimus; an immunodeficiency condition such as a human immunodeficiency viral infection or primary immunodeficiency disease; having undergone surgery (resection, sclerotherapy, or endovascular treatment), drugs (steroids, interferon, Chinese herbs, or chemotherapeutic agents) for the target lesion within at least 8 weeks prior to the date of obtaining consent for participation in this trial, or not being able to deny the possibility of remaining effects caused by surgery; pregnant, breast-feeding, or may be pregnant, or without consent to contraception during the clinical trial period; or judged by the principal investigator/sub-investigator to be ineligible to participate in this clinical trial for other reasons.

### Treatment and evaluation

Patients with a body surface area (BSA) ≥1.0 m^2^ were administered 2 mg (2 tablets) once a day, while those with a BSA of < 1.0 m^2^ were administered 1 mg (1 tablet) once a day. Patients unable to swallow whole tablets took crushed sirolimus tablets at a dose of 1.6 mg/m^2^ once per day. The dose was adjusted so that the nadir concentration remained within 5–15 ng/ml. All patients were treated with sulfamethoxazole/trimethoprim for prevention of *Pneumocystis* pneumonia. Discontinuation criteria were as follows: hematotoxicity over grade 3 according to the Common Terminology Criteria for Adverse Events (CTCAE) V4.0, adverse event other than hematotoxicity (except hyperlipidemia) over grade 4 according to CTCAE V4.0, or other equivalent reasons as determined by the principal physician.

The primary endpoint was response rate, defined as the proportion of patients who achieved a complete response or partial response as determined by radiological examination at 6 months after initiating treatment with the trial drug. The area dimensions of lymphatic tissues or cysts demonstrated using MRI with T2 fat-saturated sequences were measured using the Digital Imaging and Communications in Medicine (DICOM) viewer (OsiriX© v.9.0; Pixmeo. Bernex, Switzerland). Quantitative analysis was automatically performed to measure the area dimensions of the lesion using the region of interest (ROI) tool. If ROIs could not be calculated because of the intricate shapes of the lesions, measurement was performed using a manual computing tool (closed polygon ROI). Other pathological lesions, namely inflammatory, bleeding, and hematomas, were removed. The volume of the target lesion was calculated by multiplying these ROI areas by the slice width. If the affected area was diffuse or extensive, the measuring range was based on the normal organ position and landmarks (e.g., location of the spine). The evaluation criteria were defined as follows: complete response (CR), disappearance of all target lesions; partial response (PR), at least a 20% decrease in volume of the target lesion; progressive disease (PD), a 20% or greater increase in volume of the target lesion; and stable disease (SD), insufficient shrinkage to qualify as a partial response and insufficient growth to qualify as PD. Secondary endpoints were the response rate at 3 months, improvement in clinical symptoms caused by LM lesions, QOL scores at pretreatment and 6 months, and side effects. These were measured using PedsQL™ 4.0 Generic Core Scales (< 25 years old) [12], Functional Assessment of Cancer Therapy-General (FACT-G) (> 25 years old) [13], and CTCAE V4.0, respectively. The QOL scale was adjusted based on the QOL scale for each age. Regarding clinical symptoms, the optimal measure of disease severity in patients with vascular anomalies has not been established because LMs cause various symptoms and affect several organs. Therefore, this study used the severity measurement score for vascular anomalies to assess the degree of impairment of affected organs (Table 1). This score has been adopted from other severity scales that have been validated or are conventional objective measurements (ex. CTCAE, World Health Organization bleeding scale [14], and modified Rankin Scale [15]).

**Table 1:** Severity scores for vascular anomalies

		Score of 0	Score of 1	Score of 2	Score of 3	Score of 4	Score of 5	Score of 6
Bleeding/hemorrhage	Low-risk organs (skin and mucosa)	None	Mild without treatment	Moderate without transfusion	Severe, transfusion indicated	Very severe with hemodynamic instability		Fatal bleeding or death
	High-risk organs (gastrointestinal tract and lungs)	None		Mild without treatment		Moderate without transfusion	Severe, transfusion indicated	Very severe or death
Thoracic lesions	Respiratory	None		Mild symptoms without treatment (PaO_2_ ≥ 80 Torr)		Chronic continuous symptoms, oxygen administration required (PaO_2_ ≥ 70 and < 80 Torr)	Severe symptoms, continuous drainage and surgery required (PaO_2_ ≥ 60 and < 70 Torr)	Very severe (PaO_2_ < 60 Torr, spO_2_ < 90%) or death
r	Cardiac	None	Very mildly symptomatic	Mildly symptomatic without treatment	Moderate, treatment required	Moderate or severe, oxygen administration required (EF < 50%)	Severe, with continuous intervention or surgery required, or heart failure (EF < 40%)	Very severe, fatal, or death
Abdominal lesions		None	Very mildly symptomatic	Moderately symptomatic without treatment	Severe, treatment required	Very severe with continuous intervention or surgery		Fatal or death
Bone lesions		None	Very mildly symptomatic	Moderately symptomatic without treatment	Severe, treatment required	Very severe with continuous intervention or surgery		Fatal or death
Cutaneous lesions		None	Very mildly symptomatic	Moderately symptomatic without treatment	Severe, treatment required	Very severe with continuous intervention or surgery		Fatal or death
Neurological symptoms		None	Very mildly symptomatic	Moderately symptomatic without treatment	Severe, treatment required	Very severe with continuous intervention or surgery		Fatal or death
Coagulation disorder and thrombocytopenia		Normal	Mild coagulation disorder or thrombocytopenia (Plt ≤100 × 10^3^/μl)	Moderate coagulation disorder, low fibrinogen levels (≤100 mg/dl), or thrombocytopenia (Plt ≤50 × 10^3^/μl)	Severe coagulation disorder, low fibrinogen levels (≤100 mg/dl), and thrombocytopenia (Plt ≤50 × 10^3^/μl); treatment required	Very severe coagulation disorder, low fibrinogen levels (≤100 mg/dl) and thrombocytopenia (Plt ≤50 × 10^3^/μl) with continuous intervention		Fatal or death
Nutritional status		Normal	Decreased oral intake or mild body weight loss (≤3%)	Moderately decreased oral intake or body weight loss (3–5%)	Poor oral intake or body weight loss (≥5%), required treatment	Very severe with continuous intervention		Fatal or death

### Data analysis

Descriptive statistical methods and the Wilcoxon signed rank test for comparison between pretreatment and 6 months were used in the statistical analyses. Statistical analysis was performed using GraphPad Prism version 7. A value of *P* < 0.05 was considered statistically significant.

## Results

### Patient characteristics

**Fig. 1:**
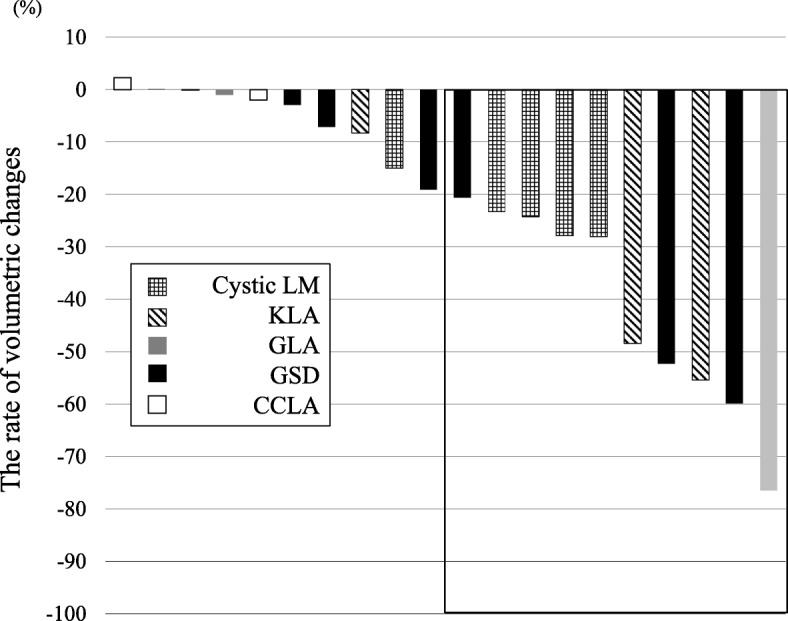
Volumetric change shown using radiological examination in patients 6 months following the start of sirolimus treatment

We reviewed 20 patients with LMs (five with cystic LM, three with KLA, three with GLA, six with GSD, and three with CCLA) who were treated with sirolimus at our institution. Patient characteristics and treatment are summarized in Table 2. Mean patient age was 16.0 years (range: 2 weeks–55 years). Five patients with LM had giant craniocervical lesions, and treatment of these lesions with surgery and sclerotherapy was not effective. They suffered from recurrence of respiratory distress, mucosal bleeding, and chronic infections. Three patients with KLA had medullary bone osteolysis, thoracic and mediastinal masses, and coagulation disorders, causing respiratory distress, chylothorax, and gastrointestinal hemorrhaging. They were pathologically diagnosed with KLA. It was difficult to control these symptoms with conventional treatment. Three patients with GLA also had thoracic and abdominal lesions and suffered from ascites, lymphorrhea, and cellulitis. Patients with GSD had osteolytic lesions in the thigh, lower limbs, skull base, and mandible. These lesions were progressive and destructive, and patients suffered from pathological fractures, pain, and neurological disorders. An infiltrative soft tissue abnormality adjacent to the area of osseous involvement was identified. Two patients with CCLA had an abnormality in the central conducting lymphatic channels, thoracic and mediastinal lesions, and leakage of lymph fluid. These symptoms were intractable and uncontrollable by conventional therapies. All patients had at least one target lesion (e.g., a cystic LM or lymphedema) that was measurable using MRI, and they were examined at 3 and 6 months (Fig. 1).

**Table 2:** Characteristics of patients treated with sirolimus

N	Age at start/ sex	Diagnosis	Location of target lesions	Complications	Previous treatment	Dosing period (months)	Range (mean) of trough concentrations (ng/mL)	Evaluation of radiological volumetric change (change rate, %)	Adverse effects associated with sirolimus (CTCAE Grade)
1	1 year/M	Cystic LM	Neck and tongue	Disturbance of swallowing, dysarthria, lymphorrhea, and airway obstruction	Sclerotherapy, Chinese herb	18 (cessation for surgery)	6.0–11.8 (7.6)	PR (−28.1)	Upper respiratory infection (3)
2	2 weeks/F	Cystic LM	Right neck, axilla, trunk, and abdominal cavity	Anemia, coagulation disorder, and bleeding	Blood transfusion	14 (cessation)	3.5–11.1 (6.2)	PR (−27.9)	None
3	3 years/F	Cystic LM	Left orbit	Ocular displacement	Steroids and propranolol	6	5.4–11.0 (8.3)	PR (−24.3)	None
4	10 months/F	Cystic LM	Neck and mediastinal	Airway obstruction and disturbance of swallowing	Sclerotherapy, Chinese herb	6	3.5–11.5 (7.2)	PR (−23.4)	Cellulitis (3)
5	11 years/M	Cystic LM	Neck and mediastinal	Airway obstruction and disturbance of swallowing	Sclerotherapy, Chinese herb	6	1.8–5.9 (3.9)	SD (−15.0)	Stomatitis (1)
6	8 years/M	KLA	Bone, thoracic and mediastinal	Chylothorax and coagulation disorder	Interferon and propranolol	30 (cessation)	4.4–9.0 (7.5)	PR (−55.4)	Pneumonia (3)
7	8 years/M	KLA	Bone, thoracic and mediastinal	Scoliosis, chylothorax and coagulation disorder	Steroids and propranolol	24	8.1–12.4 (11.2)	SD (−8.3)	Stomatitis (1)
8	20 years/M	KLA	Bone, thoracic and right chest wall	Gastrointestinal hemorrhage and coagulation disorder	Steroids	12	4.7–6.0 (5.5)	PR (−48.4)	Stomatitis (1)
9	32 years/F	GLA	Abdominal cavity and skin	Ascites, coagulation disorder, and lymphorrhea	Chinese herb	6	3.1–9.8 (6.2)	PR (−76.5)	None
10	13 years/F	GLA	Left upper limb, spleen and skin	Lymphorrhea, bleeding and cellulitis	Surgery, Chinese herb	8	2.7–7.2 (5.0)	SD (−0.1)	Stomatitis (1)
11	35 years/F	GLA	Abdominal cavity and skin	Lymphorrhea, pain and cellulitis	Surgery, Chinese herb	7 (cessation)	8.3–13.8 (11.0)	SD (−1.0)	Stomatitis (1)
12	20 years/F	GSD	Right thigh bone	Pathological fracture and pain	Surgery	18 (cessation)	3.2–10.0 (5.8)	PR (− 59.9)	Stomatitis (1)
13	9 years/M	GSD	Skull base	Hearing loss and spinal fluid leakage	Bisphosphonate, interferon, and propranolol	18 (cessation)	3.6–6.2 (5.7)	SD (−19.1)	Feeling of fatigue (1)
14	20 years/M	GSD	Right lower limbs	Lymphorrhea and spinal nerve palsy	Bisphosphonate, interferon, epidural blood patch therapy	6 (cessation)	3.0–10.9 (6.9)	PR (−20.6)	Cellulitis (3)
15	39 years/M	GSD	Paranasal sinus	Facial nerve palsy, pain, cerebral infarction, skull base osteomyelitis, and lateral medullary syndrome	Bisphosphonate and radiotherapy	6 (death from progression of disease)	3.5–7.4 (6.7)	SD (−7.1)	Stomatitis (1)
16	22 years/M	GSD	Right mandible	Spinal fluid leakage, pain, and mal interdigitation	Surgery	7	3.5–11.6 (7.0)	PR (−52.3)	None
17	18 years/M	GSD	Right thigh, lower limbs	Lymphorrhea and pain	Surgery, Bisphosphonate, interferon, Chinese herb, and propranolol	14	3.2–5.1 (4.2)	SD (−2.9)	Stomatitis (1)
18	3 years/F	CCLA	Thoracic and mediastinal	Appendicular lymphedema, respiratory disorder, dyspnea, and wheeze	Steroids and lymphatic venous anastomosis	24	3.1–7.6 (5.7)	SD (+ 2.3)	Pneumonia (2)
19	3 years/F	CCLA	Thoracic and mediastinal	Acute pancreatitis, chylothorax, and coagulation disorder	Steroids and octreotide	14	7.8–14.3 (10.3)	SD (−0.1)	Hyperlipidemia (2)
20	55 years/F	CCLA	Abdominal cavity and intestinal tract	Anemia, intestinal lymphangiectasia, and protein losing enteropathy	Transfusion, octreotide, albumin,	6 (death from progression of disease)	2.4–7.7 (4.8)	SD (−2.0)	Stomatitis (1)

*M* male, *F* female, *LM* lymphatic malformation, *KLA* kaposiform lymphangiomatosis, *GLA* generalized lymphatic anomaly, *CCLA* central conducting lymphatic anomaly, *PR* partial response, *SD* stable disease, *CTCAE* Common Terminology Criteria for Adverse Events

### Treatments, efficacy, and safety

The mean duration of sirolimus treatment was 12.5 months (range: 6–30 months). The mean trough concentration of sirolimus was 6.8 ng/ml. Although the trough level of sirolimus in 70.0% (14/20) of patients at 2 weeks after administration was less than 5 ng/ml, the levels after loading increased to target trough levels. All patients were able to continue treatment for over 6 months without any discontinuations.

**Fig. 2:**
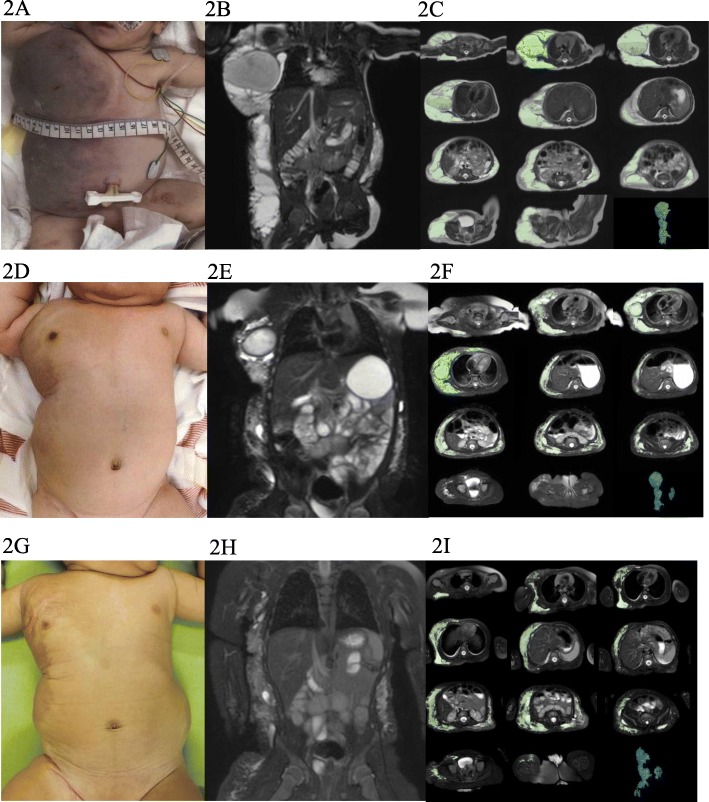
Clinical photograph, MRI, and volumetric measurements for case number 2. A 2-week-old girl had a giant cystic LM lesion in her right neck, axilla, trunk, and abdominal cavity. **a**-**c** Pretreatment. **d**-**f** 3 months after administration of sirolimus. **g**-**i** 6 months after administration of sirolimus. **c**, **f**, and **i** show volumetric measurements evaluated using the Digital Imaging and Communications in Medicine (DICOM) viewer (OsiriX)

Case number 2: a 2-week-old girl had a giant cystic LM lesion in her right neck, axilla, trunk, and abdominal cavity from the fetal period. The right axilla lesion showed hemorrhagic cysts and contained venous components. She needed a blood transfusion because of her anemia. Written informed consent was obtained from the patient’s parents for treatment and photographs and she was treated with oral sirolimus. One week later, her anemia and the color tones of her lesions improved without any other treatments, such that she did not require another blood transfusion. All LM lesions gradually decreased and the patient had no treatment-related side effects. The rate of radiological volumetric change in the lesion at 6 months was − 24.2% (Fig. 2).

**Fig. 3:**
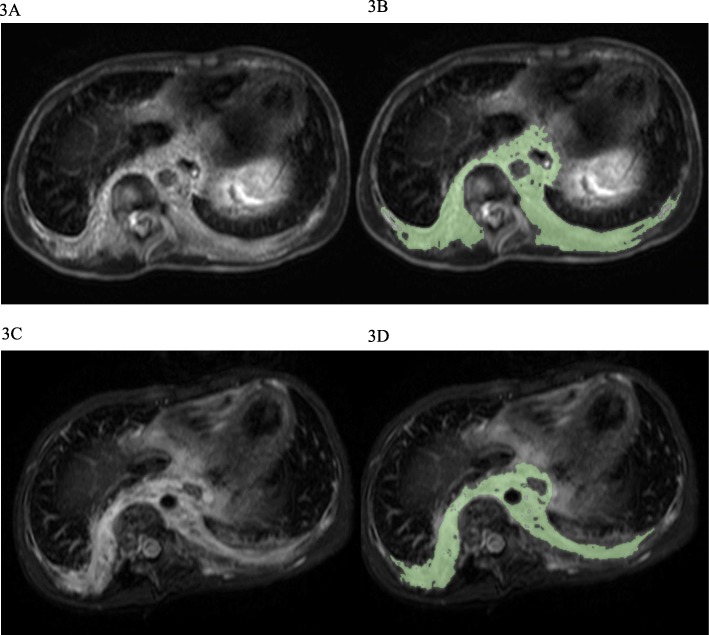
MRI and volumetric measurements for case number 7. An 8-year-old boy with KLA suffered from severe scoliosis, pain, thrombocytopenia, and coagulopathy. T2-weighted MRI of the chest demonstrates diffuse thickening of the interlobular septa and retroperitoneal soft tissue mass, which is the target lesion. **a** and **b** Pretreatment. **c** and **d** 6 months after administration of sirolimus. **b** and **d** show the volumetric measurements evaluated using the Digital Imaging and Communications in Medicine (DICOM) viewer (OsiriX)

Case number 7: an 8-year-old boy with KLA suffered from severe scoliosis, pain, thrombocytopenia, and coagulopathy. The radiological examination showed diffuse osteolytic lesions of the spine, a mediastinal mass, and thickening of bronchovascular bundles and interlobular septa. A biopsy specimen from the lesion showed dilated malformed lymphatic channels lined with a single layer of endothelial cells and foci of abnormal spindle lymphatic endothelial cells. Written informed consent was obtained from the patient’s parents for treatment and photographs and he was treated with sirolimus; however, the radiological response showed that he had SD (− 8.3% volumetric reduction in the lesion), and his symptoms, including scoliosis and coagulopathy, did not improve at 6 months after the start of treatment (Fig. 3). The patient did not have any severe side effects.

Fifty percent (10/20) of patients had a PR by radiological examination at 6 months. At 3 months, 35.0% (7/20) already showed a PR and the volume of their lesions was reduced over time. No patient achieved a CR. Lesion size did not increase by 20% or more in any patient; however, symptoms of one GSD patient (number 15) and one CCLA patient (number 20) worsened, and both died because of disease progression. The entire patient cohort had a significant improvement in the total severity score of their disease and in the QOL score (*p* = 0.0029 and *p* = 0.0129, respectively) (Table 3). Both physical and psychological QOL scores also improved. The PR group of patients showed a significant improvement in the total severity score of their disease and QOL score (*p* = 0.0020 and *p* = 0.0117, respectively). However, 10 patients who had SD and did not have a reduction in the size of their lesions showed no improvement in the total severity score or QOL score. Four SD patients (numbers 5, 10, 18, and 19) did not have any reduction in the size of their lesions, but total severity scores and QOL scores improved. Some severity scores of particular organs in patients with symptoms at pretreatment showed a tendency of improvement (the mean change severity score after sirolimus treatment: bleeding of low-risk organ (*n* = 8); − 1.125, respiratory (*n* = 9); − 1.0, abdominal lesions (*n* = 6); − 1.0, cutaneous lesions (*n* = 12); − 0.6667) (Additional file 1.

**Table 3:** Severity and QOL scores during sirolimus treatment

	Severity score			QOL score								
				All score			Physical score			Psychological score		
	Pretreatment	6 months	*p* value^a^	Pretreatment	6 months	*p* value^a^	Pretreatment	6 months	*p* value^a^	Pretreatment	6 months	*p* value^a^
All patients (*n* = 20)	8.0 (3–20)	5.0 (1–20)	0.0029	58.1 (17.4–94.6)	77.3 (7.7–91.3)	0.0129	50.0 (0–100)	77.6 (0–100)	0.0105	65.0 (12.5–100)	75.8(16.7–100)	0.0052
PR patients (*n* = 10)	8.0 (3–13)	4.5 (1–7)	0.0020	58.1 (17.4–84.7)	76.2 (51.1–91.3)	0.0117	48.4 (18.8–98.4)	77.6 (21.9–98.4)	0.0078	65.0 (12.5–78.1)	75.8 (60–90.5)	0.0156
SD patients (*n* = 10)	6.5 (3–20)	5.5 (1–20)	0.4375	63.3 (23.9–94.6)	79.8 (7.7–91.3)	0.3828	53.1 (0–100)	69.5 (0–100)	0.367	67.5 (27.5–-100)	82.5 (16.7–100)	0.2188

Values presented as median (range)

*PR* partial response, *SD* stable disease

^a^Wilcoxon signed rank test for comparison between pretreatment and 6 months

Among the 20 patients, 55.0% (11/20) remain currently under treatment; however, 30.0% (6/20) stopped treatment because their symptoms improved or did not improve (No. 11). Symptoms in one patient with GSD (No. 12) relapsed after discontinuation of sirolimus; sirolimus treatment was resumed in that case. Eighty percent of patients (16/20) had side effects, such as stomatitis, infection, and hyperlipidemia. Grade 3 infections (upper respiratory infection, cellulitis, and pneumonia) associated with sirolimus were seen in three patients, but no patient discontinued treatment, and sirolimus was generally well tolerated.

## Discussion

In this study, we analyzed the effects of sirolimus for the treatment of LMs. Our study protocol used novel and facile methods of radiological examination. We assessed the association between radiological response and clinical symptom improvements. Patients that had a reduction in the size of the lymphatic area affected showed an improvement in both clinical symptoms and QOL scores. Assessments of QOL and severity measurement scores were useful in evaluating the efficacy of sirolimus treatment.

Sirolimus has recently been reported to be effective in the treatment of vascular anomalies [16, 17]. It has been shown to be highly efficacious in improving the condition of LM patients [17]. In a recent review, 95.2% (60/63) of patients reported in previous studies showed some response to sirolimus treatment [17]. They included not only patients with LMs but also those with capillary-lymphatico-venous malformations and venolymphatic malformations. In patients with LMs, the response rate was 92.1% (35/38, excluding seven patients who were not reported), with three patients having PD. Although we have to consider publication bias, the response rate in this review was extremely high. Their review included a heterogeneous patient population that was similar to the patients in our study. If our response criteria included not only the radiological response but also severity and QOL scores, then the response rate would increase to 70% (14/20), hence exceeding the radiological response rate alone. These results were also similar to those of previous reports. Most patients had a partial response, not a complete response [17], but sirolimus may be a very useful option for the treatment of patients with LMs. For example, the ability of sirolimus to shrink LM lesions may make surgical resection possible in more patients, and sirolimus could play a role in the preoperative treatment for refractory cases of massive LM lesions.

Methods for evaluating the efficacy of sirolimus treatment have not been established. Because LM patients have variable symptoms and problems, we need to establish objective and comprehensive methods. A previous phase II trial reported using three distinct assessments involving radiological examination, functional impairment score, and QOL score [6]. Scoring functional impairment has never been validated for quantification of LAs. Our primary endpoint was radiological volumetric change at 6 months because this parameter is the most objective one amongst various other parameters. We evaluated not only the clinical symptoms but also severity and QOL scores. We referenced common criteria of each organ and dysfunctions because these criteria were very useful and easy for us to assess [14, 15]. Our results revealed that severity scores could be used to assess treatment efficacy, but we used only the sum score and not each individual symptom score in the statistical analysis because of differences in symptoms experienced by each patient. We also analyzed the change in score for each organ in our patients (see Additional file 1), which showed a tendency for improvement in each organ. Unfortunately, these data were insufficient to examine the efficacy because the number of cases was small. However, each score might be able to be used in evaluating the severity of each symptom because these scores have already been used as criteria for assessing each organ in various studies. Furthermore, we used QOL scores and these were very useful in evaluating patients. It is difficult to do QOL surveys of pediatric patients because we must consider the development and age of the child as well as the specific disease. PedsQL Scales were used in a previous study [6] and have been shown to have high versatility in a variety of diseases and situations. It has been found that there is a significant relationship between observed radiological reduction in lesions and improvement in PedsQL scores. Our results showed that volume reduction in lesions could lead to improvements in a patient’s state.

Precise assessment of radiological images of LAs is very important. No standardized methods for evaluating LAs have been reported. We used MRI to assess volumetric changes in lesions because MRI can be used to assess soft tissues with high resolution and without radiation exposure and is often used to evaluate vascular anomalies. The DICOM viewer OsiriX is an easy-to-use open-source software that allows measurement of the area of a lesion with the ROI tool. Lymphatic lesions, including lymphedema and lymphatic cysts as well as lymphatic fluids, are detectable on T2 fat-saturated images as high-intensity areas. Using this methodology, we could readily evaluate our patients’ images. Thus, in this study, we used simple and novel methods for evaluating LMs.

LAs result from defects in the lymphangiogenesis, lymphatic development, and lymphatic vasculature remodeling [18]. Recent studies revealed that there were somatic genetic abnormalities in patients with LMs [7]. Sporadic LMs may be caused by somatic changes in components of the phosphoinositide 3-kinase (PI3K)/mTOR and RAS/mitogen-activated protein (MAPK) signaling pathways [19]. MTOR inhibitors target protein synthesis downstream of the Akt pathway and are predicted to be effective in disorders where the mTOR growth control pathway is affected [19]. Somatic activation of related genes can cause the growth of abnormal lymphatic endothelial cells and dysplasia of lymphatic canals and valves [18]. This would be associated with the pathogenesis of local lymphatic dysfunction or excessive activation of local lymphatic tissues. It may occur at the normal distribution areas or from lesions that are not distributed. Although the mechanism by which sirolimus affects LMs is still unknown at present, normalization or inactivation of signaling pathways involved in the development of abnormal lesions might play a role in the efficacy of sirolimus treatment. In experimental models, sirolimus is thought to act on lymphatic tissues within lesions to regulate the production and leakage of lymph by decreasing lymphatic endothelial cell activity. In a lymphangiectasia mouse model, Baluk et al. demonstrated that sirolimus not only prevents the growth of abnormal lymphatics but also induces the partial regression of lesions, without apparent effects on normal lymphatics [20]. This regression is accompanied by reductions in Prox1 and vascular endothelial growth factor receptor-3 but not by caspase-dependent apoptosis of lymphatic endothelial cells. In our study, sirolimus caused a reduction in the volume of lymphatic lesions. This may lead to an impairment in abnormal lymphatic flow and a decreased flow of lymph fluid. However, the dilated lymphatic wall remained.

Our study had some limitations. First, this was a study and the number of registered patients was small. It included patients with a variety of LAs and heterogeneous disorders, so it was necessary to consider the differences of each disease or condition. Second, the severity scores that we used have not been validated in assessing vascular anomalies; however, these severity scores consist of general criteria for evaluating severities of each organ and condition, and we will be assessing their validity for these conditions in a future study. Third, trough levels should be maintained at 5–15 ng/ml, as shown in previous studies; however, actual trough levels ranged more widely. In a previous phase 2 study, trough levels were maintained at 10–15 ng/ml [6]. A systematic review reported that the expected trough levels of sirolimus in most studies (19/25, 76.0%) were 5–15 ng/ml [21]. Currently, there are no standardized methods for its optimal dosing. A previous in vitro study demonstrated that the highest dose of rapamycin reduced the incidence of lymphatic anomalies, but it also increased toxicities [20]. There were some patients who achieved good responses with trough levels less than 5 ng/ml. Further study of the association between trough levels and efficacy and safety of sirolimus is needed.

In conclusion, we performed a prospective study of sirolimus treatment in patients with LMs and employed useful assessment methods. Sirolimus reduces the lymphatic tissue volume associated with LMs and could lead to improvement of clinical symptoms and QOL.

## Additional file

Additional file 1: Changes in severity score after sirolimus treatment. (DOCX 33 kb)

## Data Availability

The datasets and analysis performed during the current study are available from the corresponding author upon reasonable request.

## Abbreviations

BSA: Body surface area
CCLA: Central conducting lymphatic anomaly
CR: Complete response
CTCAE: Common Terminology Criteria for Adverse Events
DICOM: Digital Imaging and Communications in Medicine
FACT-G: Functional Assessment of Cancer Therapy-General
GLA: Generalized lymphatic anomaly
GSD: Gorham-Stout disease
ISSVA: International Society for the Study of Vascular Anomalies
KLA: Kaposiform lymphangiomatosis
LA: Lymphatic anomaly
LM: Lymphatic malformation
MRI: Magnetic resonance imaging
mTOR: mammalian target of rapamycin
PD: Progressive disease
PR: Partial response
QOL: Quality of life
ROI: Region of interest
SD: Stable disease
