# Supplementary material for: The impact of sirolimus therapy on lesion size, clinical symptoms, and quality of life of patients with lymphatic anomalies
Source: Orphanet J Rare Dis. 2019 Jun 13;14:141. doi: 10.1186/s13023-019-1118-1 (PMC6567608; doi:10.1186/s13023-019-1118-1)
Supplement: Supplementary file 1 — Changes in severity score after sirolimus treatment. (DOCX 33 kb) [file 13023_2019_1118_MOESM1_ESM.docx]

**Additional file 1. Changes in severity score after sirolimus treatment**

1) Bleeding in low-risk organs (skin and mucosa）

|  | | 6 months after the start of administration of sirolimus | | | | | | |
| --- | --- | --- | --- | --- | --- | --- | --- | --- |
|  |  | Score of 0 | Score of 1 | Score of 2 | Score of 3 | Score of 4 | Score of 5 | Score of 6 |
| Pretreatment | Score of 0 |  |  |  |  |  |  |  |
|  | Score of 1 | 14 (PR) | 10 (SD), 11 (SD), 15 (SD) |  |  |  |  |  |
|  | Score of 2 |  | 1 (PR) | 6 (PR) |  |  |  |  |
|  | Score of 3 | 2 (PR) |  |  |  |  |  |  |
|  | Score of 4 | 4 (PR) |  |  |  |  |  |  |
|  | Score of 5 |  |  |  |  |  |  |  |
|  | Score of 6 |  |  |  |  |  |  |  |

Mean: -1.125 (95% confidence interval = -2.423–0.173)

2) Bleeding in high-risk organs (gastrointestinal tract and lungs)

|  | | 6 months after the start of administration of sirolimus | | | | | | |
| --- | --- | --- | --- | --- | --- | --- | --- | --- |
|  |  | Score of 0 | Score of 1 | Score of 2 | Score of 3 | Score of 4 | Score of 5 | Score of 6 |
| Pretreatment | Score of 0 |  |  |  |  |  |  |  |
|  | Score of 1 |  |  |  |  |  |  |  |
|  | Score of 2 |  |  |  |  |  |  |  |
|  | Score of 3 | 8 (PR) |  |  |  |  |  |  |
|  | Score of 4 |  |  |  |  |  |  |  |
|  | Score of 5 |  |  |  |  |  | 20 (SD) |  |
|  | Score of 6 |  |  |  |  |  |  |  |

3) Thoracic lesions (respiratory)

|  | | 6 months after the start of administration of sirolimus | | | | | | |
| --- | --- | --- | --- | --- | --- | --- | --- | --- |
|  |  | Score of 0 | Score of 1 | Score of 2 | Score of 3 | Score of 4 | Score of 5 | Score of 6 |
| Pretreatment | Score of 0 |  |  |  |  |  |  |  |
|  | Score of 1 |  |  |  |  |  |  |  |
|  | Score of 2 |  |  | 5 (SD), 7 (SD), 20 (SD) |  |  |  |  |
|  | Score of 3 |  |  | 4 (PR) |  |  |  |  |
|  | Score of 4 |  |  | 1 (PR), 6 (PR), 8 (PR), 19 (SD) |  | 18 (SD), |  |  |
|  | Score of 5 |  |  |  |  |  |  |  |
|  | Score of 6 |  |  |  |  |  |  |  |

Mean: -1.0 (95% confidence interval = -1.769 to -0.2313)

4) Thoracic lesions (cardiac)

|  | | 6 months after the start of administration of sirolimus | | | | | | |
| --- | --- | --- | --- | --- | --- | --- | --- | --- |
|  |  | Score of 0 | Score of 1 | Score of 2 | Score of 3 | Score of 4 | Score of 5 | Score of 6 |
| Pretreatment | Score of 0 |  |  |  |  |  |  |  |
|  | Score of 1 |  |  |  |  |  |  |  |
|  | Score of 2 |  |  | 19 (SD) |  |  |  |  |
|  | Score of 3 |  |  |  |  |  |  |  |
|  | Score of 4 |  |  |  |  |  |  |  |
|  | Score of 5 |  |  |  |  |  |  |  |
|  | Score of 6 |  |  |  |  |  |  |  |

5) Abdominal lesions

|  | | 6 months after the start of administration of sirolimus | | | | | | |
| --- | --- | --- | --- | --- | --- | --- | --- | --- |
|  |  | Score of 0 | Score of 1 | Score of 2 | Score of 3 | Score of 4 | Score of 5 | Score of 6 |
| Pretreatment | Score of 0 |  |  |  |  |  |  |  |
|  | Score of 1 |  |  |  |  |  |  |  |
|  | Score of 2 | 8 (PR) |  | 11 (SD) |  |  |  |  |
|  | Score of 3 |  | 9 (PR) | 3 (PR) |  |  |  |  |
|  | Score of 4 |  |  |  | 18 (SD) | 20 (SD) |  |  |
|  | Score of 5 |  |  |  |  |  |  |  |
|  | Score of 6 |  |  |  |  |  |  |  |

Mean: -1.0 (95% confidence interval = -1.939 to -0.06136)

6) Bone lesions

|  | | 6 months after the start of administration of sirolimus | | | | | | |
| --- | --- | --- | --- | --- | --- | --- | --- | --- |
|  |  | Score of 0 | Score of 1 | Score of 2 | Score of 3 | Score of 4 | Score of 5 | Score of 6 |
| Pretreatment | Score of 0 |  |  |  |  |  |  |  |
|  | Score of 1 |  | 4 (PR), 6 (PR), 7 (PR), 8 (PR), |  |  |  |  |  |
|  | Score of 2 | 11 (SD) | 16 (PR) | 13 (SD), 17 (SD) |  |  |  |  |
|  | Score of 3 |  |  |  | 12 (PR), 14 (SD), 20 (SD) |  |  | 15 (SD) |
|  | Score of 4 |  |  |  |  |  |  |  |
|  | Score of 5 |  |  |  |  |  |  |  |
|  | Score of 6 |  |  |  |  |  |  |  |

Mean: 0 (95% confidence interval = -0.7168–0.7168)

7) Cutaneous lesions

|  | | 6 months after the start of administration of sirolimus | | | | | | |
| --- | --- | --- | --- | --- | --- | --- | --- | --- |
|  |  | Score of 0 | Score of 1 | Score of 2 | Score of 3 | Score of 4 | Score of 5 | Score of 6 |
| Pretreatment | Score of 0 |  |  |  |  |  |  |  |
|  | Score of 1 |  | 4 (PR), 17 (SD), 20 (SD) |  |  |  |  |  |
|  | Score of 2 |  | 1 (PR), 2 (PR), 3 (PR), 5 (SD), 8 (PR), 10 (PR), 14 (PR) | 19 (SD) |  |  |  |  |
|  | Score of 3 |  |  | 11 (PR) |  |  |  |  |
|  | Score of 4 |  |  |  |  |  |  |  |
|  | Score of 5 |  |  |  |  |  |  |  |
|  | Score of 6 |  |  |  |  |  |  |  |

Mean: -0.6667 (95% confidence interval = -0.9795–0.3538)

8) Neurological symptoms

|  | | 6 months after the start of administration of sirolimus | | | | | | |
| --- | --- | --- | --- | --- | --- | --- | --- | --- |
|  |  | Score of 0 | Score of 1 | Score of 2 | Score of 3 | Score of 4 | Score of 5 | Score of 6 |
| Pretreatment | Score of 0 |  |  |  |  |  |  |  |
|  | Score of 1 |  | 4 (PR), 16 (PR), 17 (SD) |  |  |  |  |  |
|  | Score of 2 |  |  | 13 (SD) |  |  |  |  |
|  | Score of 3 |  | 3 (PR) |  | 14 (SD) |  |  |  |
|  | Score of 4 |  |  |  |  | 15 (SD) |  |  |
|  | Score of 5 |  |  |  |  |  |  |  |
|  | Score of 6 |  |  |  |  |  |  |  |

Mean: -0.2857 (95% confidence interval = -0.9848–0.4134)

9) Coagulation disorder and thrombocytopenia

|  | | 6 months after the start of administration of sirolimus | | | | | | |
| --- | --- | --- | --- | --- | --- | --- | --- | --- |
|  |  | Score of 0 | Score of 1 | Score of 2 | Score of 3 | Score of 4 | Score of 5 | Score of 6 |
| Pretreatment | Score of 0 |  |  |  |  |  |  |  |
|  | Score of 1 | 5 (SD) | 6 (PR), 8 (SD), 11 (SD), 20 (SD) |  |  |  |  |  |
|  | Score of 2 |  |  | 7 (SD) |  |  |  |  |
|  | Score of 3 |  |  |  |  |  |  |  |
|  | Score of 4 |  |  |  |  |  |  |  |
|  | Score of 5 |  |  |  |  |  |  |  |
|  | Score of 6 |  |  |  |  |  |  |  |

Mean: -0.1667 (95% confidence interval = -0.5951–0.2618)

10) Nutritional status

|  | | 6 months after the start of administration of sirolimus | | | | | | |
| --- | --- | --- | --- | --- | --- | --- | --- | --- |
|  |  | Score of 0 | Score of 1 | Score of 2 | Score of 3 | Score of 4 | Score of 5 | Score of 6 |
| Pretreatment | Score of 0 |  |  |  |  |  |  |  |
|  | Score of 1 |  | 1 (PR), 4 (PR) |  |  |  |  |  |
|  | Score of 2 |  |  |  |  |  |  |  |
|  | Score of 3 |  |  |  |  | 15 (SD) |  |  |
|  | Score of 4 |  |  |  |  | 18 (SD), 20 (SD) |  |  |
|  | Score of 5 |  |  |  |  |  |  |  |
|  | Score of 6 |  |  |  |  |  |  |  |

Mean: 0.2 (95% confidence interval = -0.3553–0.7553)

This table shows the changes in severity score for each organ from pretreatment to 6 months after the start of administration of sirolimus. The number indicates patient number. Patients who did not have symptoms (score 0) at pretreatment were not included in this table because their scores did not change at 6 months after the start of administration of sirolimus. PR: partial response, SD: stable disease
